# Attributes Influencing Willingness to Pay for Overweight and Obesity Interventions in Adults: A Systematic Literature Review

**DOI:** 10.7759/cureus.65468

**Published:** 2024-07-26

**Authors:** Nurul A Anwar, Azimatun Noor Aizuddin, Norfazilah Ahmad, Abdul Aziz

**Affiliations:** 1 Epidemiology and Public Health, Universiti Kebangsaan Malaysia Medical Centre, Kuala Lumpur, MYS; 2 Public Health Medicine, Faculty of Medicine, Universiti Kebangsaan Malaysia, Kuala Lumpur, MYS; 3 Public Health Medicine, Universiti Kebangsaan Malaysia, Kuala Lumpur, MYS; 4 Public Health, Ministry of Health Malaysia, Putrajaya, MYS

**Keywords:** systematic literature review, willingness to pay (wtp), overweight, obesity, intervention

## Abstract

This review aims to identify the associated attributes of willingness to pay (WTP) for overweight and obesity interventions. A narrative review was conducted by partially adopting the Preferred Reporting Items for Systematic Reviews and Meta-Analyses (PRISMA) guideline. A non-exhaustive search using a pre-defined strategy and keywords was done on three selected literature databases: Pubmed, Scopus, and Web of Science. The inclusion criteria for the review were original studies written in English, published between 2000 and 2022, and focused on WTP for overweight and obesity interventions in adults. The extracted studies were manually screened for their eligibility through three cascading tiers: the title, the abstract, and the full article. Only nine original studies were eligible for review based on the screening procedure of 40 screened articles. There was heterogeneity in the study designs, methods, target populations, study duration, and perspectives across the studies. The majority of the studies showed that higher WTP was associated with younger age, having higher income, being female, having higher body mass index (BMI), having the perception of being overweight, habits, and attitudes. WTP is also attributed to the associated percentage of weight loss, long-term health risk reduction, time to noticeable weight loss, delivery mode, side effects, lifestyle modification, and costs of interventions. The identification of common attributes of the WTP for overweight and obesity intervention can assist in the formulation and implementation of effective evidence-based policies. Specific sub-groups with low WTP could be targeted via unique initiatives to improve their participation in weight-loss interventions.

## Introduction and background

In recent decades, obesity has grown significantly as a global health concern, with its prevalence and incidence rates rising worldwide [1]. In 2020, 2.6 billion of the global population were overweight (BMI ≥ 25 kg/m^2^), whereby this figure is projected to increase to four billion people by 2035 [2]. The prevalence of obesity (BMI ≥ 30 kg/m^2^) alone is anticipated to rise from 14% to 24% of the global population over the same period, affecting nearly two billion adults, children, and adolescents by 2035 [2]. This is a major public health crisis, as many diseases, including diabetes, heart disease, and various forms of cancer, are linked to obesity. Moreover, it poses a significant risk for adverse coronavirus disease 2019 (COVID-19) outcomes [1].

Healthcare systems face ever-increasing costs due to the rising prevalence of obesity and its related diseases. Taxpayers and governments often bear these costs, which can strain each nation’s healthcare budget. The estimated global cost of obesity-related health problems and loss of productivity in 2016 was approximately USD 2 trillion [2]. This figure shows how urgently the worldwide obesity crisis needs to be dealt with by means of effective public health initiatives, laws, and personal behavior changes [1].

The best strategy to reduce the cost burden of obesity-related diseases, both financially and medically, is through interventions concentrating on adequate structural infrastructure to support a healthy lifestyle, public awareness, and education [3]. Comprehensive weight management interventions should include public education campaigns, subsidies for healthy foods, physical activities, and possible incentives for individuals who achieve and maintain a healthy weight.

Willingness to pay (WTP) may point to a potential solution in implementing policies encouraging individuals to take responsibility for their health, including weight loss [4]. In the context of this review, WTP is the amount of money a person is willing to spend to meet their desired weight loss. WTP for the interventions of obesity and overweight is a complicated issue that differs individually and is affected by various attributes. For example, due to the diversity of personal beliefs, some individuals may prioritize natural or holistic approaches to weight management and may be more willing to pay for these treatments. In contrast, others may prefer interventions that are more conventional [5]. Evidence-based suggestions for decision-makers and policymakers may be a wise course of action to address the heterogeneity of these attributes [6].

The decision to implement policies to address the burden of obesity on healthcare systems is complex and requires careful consideration of the various attributes, including the individual characteristics in WTP for weight management interventions. With this purpose in mind, this review aims to identify the associated attributes of WTP for overweight and obesity interventions based on the studies.

## Review

Research question formulation

The review question was developed using the "population, exposure, outcome" (PEO) concept. As this review focuses on the relationship between certain attributes and associated health outcomes, the use of the PEO concept is recommended. In this systematic review, population refers to the general population, exposure refers to associated attributes, and outcome is the WTP for overweight and obesity interventions. The review question is what are the attributes associated with the WTP for obesity intervention? The keywords used are presented in Table 1.

**Table 1:** Keywords used in the screening process of the database

Databases Search String
Scopus 1) TITLE-ABS-KEY(("willingness to pay" ") AND ("obesity intervention" OR "overweight intervention ")
Web of Science 1) TS =) (("willingness to pay" ") AND ("obesity intervention" OR "overweight intervention ") PubMed
PubMed 1)(("willingness to pay" ") AND ("obesity intervention" OR "overweight intervention ")

Selection procedure

A systematic literature search based on the Preferred Reporting Items for Systematic Reviews and Meta-Analyses (PRISMA) guidelines [7] was conducted to retrieve published original studies written in English that focused on WTP for overweight and obesity interventions. The electronic search was performed using three chosen literature databases: Pubmed, Scopus, and Web of Science. Only articles published between 2000 and 2022 were included in the review. The pre-defined Boolean search strategy employed for the database search included the following terms: "overweight," "obesity," "interventions," and “willingness to pay." Studies included in this review focused on WTP studies for overweight and obesity interventions of any relevant design that examined the associated attributes of the WTP.

**Figure 1:**
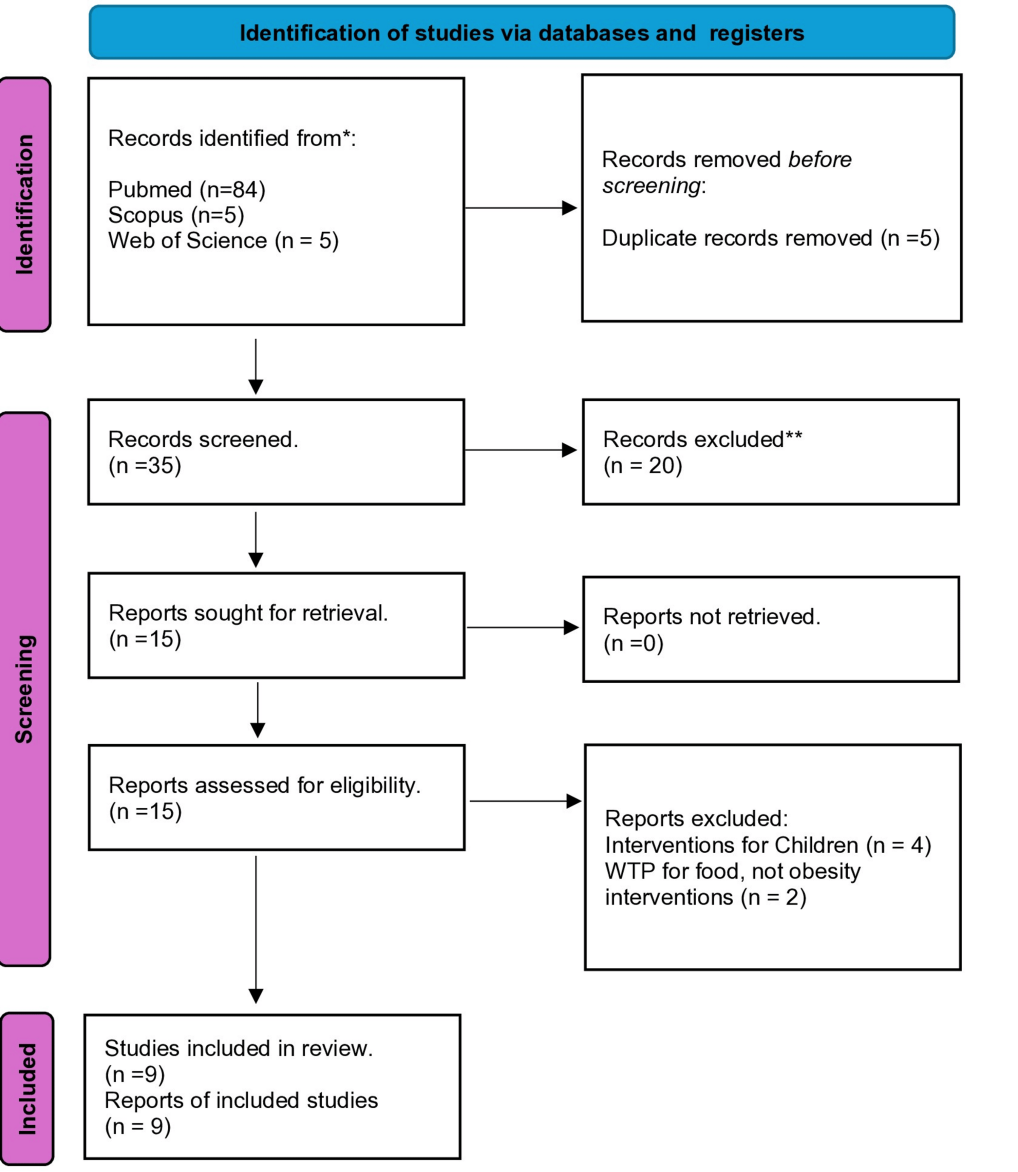
Flowchart of the study selection processes

The inclusion criteria were the following: (1) publication from 2000 to 2022; (2) publication in the English language; and (3) original article. These attributes include socio-demographic characteristics, socioeconomic status, and other relevant independent factors. Studies outside the scope of the review objective were excluded from the review. The procedure is shown in Figure 1.

Data extraction and synthesis

Data extraction of the following information was done by standardized data extraction on all nine studies to be included in the evidence table. Information collected includes (1) author, (2) publication year, (3) reference/title, (4) country of study location, (5) method, (6) sample size, (7) results, and (8) relevant WTP attributes. Data synthesis was achieved using content analysis in order to condense text into fewer content-related categories.

Critical appraisal of the included studies

The studies were appraised using the Mixed Methods Appraisal Tool (MMAT). The MMAT is a critical appraisal tool that is developed to appraise studies included in systematic mixed study reviews. The methodology quality of five categories of studies (qualitative study, randomized control trials, non-randomized studies, quantitative descriptive study, and mixed methods study) can be appraised using this tool. For each category, five criteria are used to assess the quality of the study. The suggestion was provided for reporting an overall score (5*****/100% quality criteria met; 4****/80% quality criteria met; 3***/60% quality criteria met; 2**/40% quality criteria met; 1*/20% quality criteria met). Of the included studies, one study was given a score of 80%, while the other eight studies were given a score of 100%. The scores of MMAT for each included study are shown in Table 2.

**Table 2:** Critical appraisal of the selected studies using the Mixed Methods Appraisal Tool (MMAT)

No	Author, Year	The Mixed Methods Appraisal Tool Criteria																									Quality (%)
		1.1	1.2	1.3	1.4	1.5	2.1	2.2	2.3	2.4	2.5	3.1	3.2	3.3	3.4	3.5	4.1	4.2	4.3	4.4	4.5	5.1	5.2	5.3	5.4	5.5	
1	Fu et al., 2011 [8]																yes	yes	yes	yes	yes						100%
2	Liu et al., 2018 [9]																yes	yes	yes	yes	yes						100%
3	Sayruamyat, 2018 [10]																yes	yes	yes	yes	yes						100%
4	Narbro et al., 2000 [11]																yes	yes	yes	yes	yes						100%
5	Ahmed et al., 2020 [12]																yes	No	yes	yes	yes						80%
6	de-Magistris et al., 2016 [13]																yes	yes	yes	yes	yes						100%
7	Doyle et al., 2012 [14]																yes	yes	yes	yes	yes						100%
8	Khawali et al., 2014 [15]																yes	yes	yes	yes	yes						100%
9	Jerome et al., 2015 [16]																yes	no	yes	yes	yes						100%

Discussion

Based on the current review, it was demonstrated that certain attributes and individual differences could influence a person's propensity to pay for weight loss interventions. This WTP can vary significantly based on various sociodemographic factors such as gender, age, income, education, ethnicity, and beliefs. Other factors such as BMI and co-morbidities also play a significant role. WTP is also attributed to the associated percentage of weight loss, long-term health risk reduction, time to noticeable weight loss, delivery mode, side effects, lifestyle modification, and costs of interventions The pattern of these factors across the HIC and MIC was nearly similar, pointing towards the universality of these attributes for WTP of weight loss interventions. The results are shown in Table 3.

**Table 3:** Evidence for the attributes of WTP BMI: body mass index; GBP: British pound sterling; kg: kilogram; PNP: Personalized Nutrition Programme; MYR: Malaysian Ringgit; NT: New Taiwan Dollar; TOT: theory of trying; THB: Thai Baht; USD: US Dollar; WTP: willingness to pay

No	Author/years/title	Objectives	Method	Sample/country	WTP	Attributes to WTP
1	Fu et al., 2011 [8]: Willingness to pay (WTP) for obesity prevention	To estimate consumer’s WTP	Contingent valuation	150/Taiwan	NT 12,531 (USD 362) for 5 kg/3 months	Higher BMI, females, higher education, higher income, perceived health, younger age
2	Liu et al., 2018 [9]: Willingness to pay for weight-control treatment	To estimate WTP for alternative forms of weight-control treatment and evaluate how it varies with individual characteristics	Contingent valuation	1,441/Taiwan	Medicine: USD 12/month, Low-calorie diet: USD 10/month	Higher income, BMI, perception, peer pressure, younger age, females
3	Sayruamyat, 2018 [10]: Impacts of attitudes and personality traits on weight goals and willingness to pay for a personalised nutrition programme in Thailand	theory of trying (TOT) to estimate WTP for a personalized nutrition programme (PNP)	Contingent valuation	597/Thailand	Maximum THB 1272/ (USD 35) per month for PNP	Attitudes, habits, younger age, higher income, single, employment, time, preference
4	Narbro et al., 2000 [11]: Willingness to pay for obesity treatment	To measure the WTP for effective treatment in obese individuals 2) to assess the relationship between WTP and parameters reflecting the severity of obesity and subjects’ socio-demographic characteristics	Contingent valuation	2,000/Sweden	Willing to pay approximately twice monthly salary for effective treatment	Higher BMI, females, higher education, higher income, perceived health, younger age
5	Ahmed et al., 2020 [12]: Estimating clinical burden and valuation of weight management strategies (using WTP) for overweight and obesity in primary care setting in Borneo Sarawak, Malaysia	To estimate the clinical burden of obese patients in government primary care clinics	Valuation	400/Malaysia	Median MYR 88.80 (USD 21.01)	Preference, delivery mode
6	de-Magistris et al., 2016 [13]: Consumers' WTP for nutritional claims in reducing the obesity epidemic	Consumers' WTP for nutritional claims in reducing the obesity epidemic	Real choice experiment	502/Spain	Consumers are willing to pay a price premium for a package of cheese with a reduced-fat claim	Higher income, working, younger age, higher education, normal weight
7	Doyle et al., 2012 [14]: Willingness to pay for obesity pharmacotherapy	A study among individuals seeking weight loss, their preferences, and WTP for obesity therapies	Discrete choice experiments	502/United Kingdom, United States	GBP 6.51/ USD 10.49 per month per percentage point of weight loss that pharmacotherapy could provide.	Percentage of weight loss, long-term health risk reduction, duration Delivery mode, side effects, lifestyle modification, cost
8	Khawali et al., 2014 [15]: Willingness to pay as patient preference for bariatric surgery	To evaluate preferences of severely obese patients for obesity surgical treatment	Contingent valuation	100/Brazil	WTP: 40% higher than family income.	Higher income, comorbidity, older age
9	Jerome et al., 2015 [16]: Willingness to pay for the continued delivery of a lifestyle-based weight loss program: The Hopkins Power Trial	WTP for the continued provision of a lifestyle-based weight loss program: The Hopkins trial	Interview administered questionnaires	234/United States of America (USA)	The median WTP for Non-Blacks was lower (USD 45/month) compared to that of Blacks. (USD 65/month)	Higher BMI females, Blacks, younger age

Female Gender

According to the work of Alsubhi et al. [17], being female was shown to influence the WTP for weight reduction products and services. This may be due to their higher interest in weight loss and willingness to invest in weight loss interventions. Generally, women are more concerned with their surroundings and may feel a greater sense of obligation to make sustainable decisions. Women may also be more likely to pay for products that promote health and well-being because they are generally more health-conscious and may place a higher value on self-care. This may be due to societal constraints on women to conform to a particular body type or a greater emphasis on appearance in women's lives. This societal pressure to meet beauty standards and body image concerns may drive women to pay more for weight loss interventions. In addition, women may have different perspectives on weight loss than men, whereby women place a greater emphasis on maintaining a healthy weight for their overall health.

Education

Education level may also impact the WTP for weight loss products and services. The higher WTP among individuals with a higher level of education may be due to their greater understanding of the positive effects of weight loss [17]. They are more likely to have a greater understanding of the overall long-term health benefits of weight loss and the impact of lifestyle factors on health and well-being. Furthermore, higher levels of education are often associated with a higher level of income, which can increase the ability and inclination to pay for weight loss products and services.

Income

As stated previously, the associated privilege of higher earnings can considerably influence an individual's WTP for weight loss interventions [18]. People with higher wages may have more disposable or discretionary income to spend on weight loss interventions such as gym memberships and personal trainers or healthier eating selections and specialized diets that are relatively more expensive. This relative wealth can influence individuals to be more willing to invest in their health and weight-loss goals. On the contrary, those with lower earnings may need more financial resources to invest in weight loss interventions. They may also have restricted access to healthy eating options, which can make weight loss more challenging. These constraints may diminish their WTP for weight loss interventions. Additionally, lower-income individuals may experience other challenges to weight loss commitments, such as limited free time due to job or caregiving responsibilities, limited access to safe and cheap exercise options, and anxieties associated with financial instability. Thus, it is crucial for weight reduction interventions to consider the financial limitations and other challenges faced by those with lower incomes to ensure that they are accessible and adaptable for all individuals, regardless of their income levels.

Body Mass Index (BMI)

People with higher BMIs are typically more willing to pay for weight loss interventions than people with lower BMIs. This might be the case because those with higher BMIs are more likely to suffer from obesity-related health issues [18] and hence might be more driven to slim down. Those with self-perceived overweight are 3.5 times more likely to use weight loss products than those who are comfortable with their current weight [12].

Age and Comorbidities

The higher WTP for weight loss interventions was also shown for older individuals based on the current review. Older individuals may be more likely to have health concerns related to their weight and may be more aware of the long-term health benefits of weight loss. This may be in line with their increasing comorbidities. In the context of weight loss interventions, comorbidities such as obesity-related diseases such as type 2 diabetes, hypertension, and cardiovascular disease can significantly influence an individual's WTP for weight loss interventions. As shown in the study by Khawali et al. [15], those with comorbidities were more inclined to pay for weight loss interventions due to the possible health advantages, together with the increased sense of urgency. Comorbidities also increase the health risks associated with obesity and can lead to increased healthcare costs. Hence, individuals with comorbidities may perceive weight loss interventions as more valuable and may be willing to pay more up-front to improve their health outcomes and quality. For them, this may be a valid strategy to reduce costs in the long term.

Culture and Ethnicities

It should be noted that certain ethnic groups may have different societal and familial emphases. They may prioritize weight loss over other health issues. For example, African Americans tend to spend more on weight loss interventions [16]. However, behavior generalization based on ethnicity may not be accurate because individuals within a group may vary in their attitudes and behaviors according to their circumstantial circumstances. Therefore, it is essential to consider other individual factors such as individual preferences, personal sociocultural values, and socioeconomic status when examining the differences in WTP for weight loss interventions between ethnic groups [17].

Effectiveness of Weight Loss

The effectiveness of interventions by weight loss percentage will be attributed to WTP [14]. The effectiveness can vary widely due to delivery mode, such as bariatric surgery has a high success rate regarding weight loss. WTP for healthier food options, gym memberships, or personal trainers can support weight loss efforts, but adherence to these choices is crucial to be effective. The perceived effectiveness of a treatment can be linked to its duration. Some may believe that longer interventions are more effective in achieving lasting results, while others may think that shorter interventions can provide rapid outcomes. Some individuals may believe in-person interventions are more effective due to the presence of professionals. In contrast, others may have confidence in the efficacy of self-guided or remote interventions.

*****Long-Term Health Risk*

The awareness of long-term health risk reduction associated with obesity intervention can be a powerful motivator for individuals to be willing to pay for these interventions [14]. It can change the perceived value of such interventions and align with individuals' goals of achieving better health and a higher quality of life while mitigating the risk of chronic diseases. As individuals understand that obesity interventions can reduce their long-term health risks, such as the risk of heart disease, diabetes, and certain cancers, they are more likely to perceive these interventions as valuable. Furthermore, they are more willing to invest in what they believe will protect their health and extend their lifespan.

By recognizing the value of preventive healthcare measures, obesity interventions can be seen as a preventive measure to reduce the likelihood of future health problems. Health education and awareness campaigns emphasizing the long-term health risks associated with obesity can improve individuals' health, and with the vision of tangible improvements in their health and reduced risk factors, they are more inclined to take proactive steps and allocate resources to address the issue and more likely to continue with the intervention.

Cost

Different delivery modes come with varying costs which can affect WTP [14]. In-person interventions involve facility fees, travel expenses, and higher professional fees. Conversely, online or self-guided interventions are more cost-effective and more appealing. People often conduct a cost-benefit analysis when considering healthcare interventions. If the long-term health benefits outweigh the costs of the intervention, they are more likely to be willing to pay. Knowing that an obesity intervention can reduce the risk of expensive and debilitating chronic conditions can make it a more attractive investment.

Long-term health risk reduction can also affect healthcare systems. Reducing the burden of obesity-related diseases can lead to lower overall healthcare costs. Governments and insurance companies may be more willing to invest in or subsidize obesity interventions when they recognize the potential for long-term cost savings. Longer-duration treatments may come with higher overall costs, which can influence willingness to pay. Individuals may be more willing to invest in treatments with shorter durations if they perceive them as more affordable and cost-effective.

Lifestyle Modification

Successful weight loss involves individual factors, behavioral changes, social support, and resource access. It is essential to consider all these factors when addressing obesity and weight management. Sustainable interventions that align with a person's lifestyle are more likely to yield long-term success. Minimum lifestyle modifications yield higher WTP as in the study of Doyle et al. [14]. Behavioral changes are crucial for successful weight loss as they require a person's commitment to lasting lifestyle changes. Emotional eating, stress, and mental health can significantly impact weight management. Thus, interventions that address these psychological factors are necessary for sustained weight loss.

Side Effects

The side effects of obesity intervention can substantially impact an individual's WTP for such intervention [14]. Balancing the potential benefits and risks and providing tailored information and support can help individuals make informed decisions about their healthcare choices. By risk-benefit assessment, individuals considering obesity treatment will typically weigh the potential benefits of weight loss against the risks of treatment. If the treatment is associated with severe or unpleasant side effects, it can deter individuals from pursuing it, potentially lowering their WTP. Some may be willing to endure certain side effects if they believe the treatment's benefits outweigh the drawbacks, while others may not tolerate any side effects. Expecting severe or life-altering side effects can significantly reduce willingness to invest in treatment. Short-term, reversible side effects may be more acceptable than long-term or permanent ones.

Duration/Time Preference

The duration of obesity interventions is crucial in influencing an individual's WTP for such interventions [10,14]. Personal goals, financial considerations, perceptions of effectiveness, and individual preferences contribute to determining the duration an individual is willing to commit. Tailoring obesity interventions to align with individual needs and preferences can enhance acceptability and WTP. People often have different goals regarding obesity treatment. Those seeking short-term results may be willing to pay for a shorter, more intense intervention, while those prioritizing long-term health may be prepared to invest in a more extended treatment program. Longer-term treatments require more time, effort, and resources and may be perceived as more challenging to sustain.

Delivery Mode

The delivery mode of obesity interventions can significantly influence an individual's WTP based on convenience, access to resources, cost, support, privacy, and personal preferences such as in Doyle et al. [14]. Some individuals may be more willing to pay for interventions that can be easily integrated into their daily lives such as online programs, mobile apps, or telemedicine-based interventions that offer convenience, allowing individuals to access support and information from the comfort of their homes.

WTP may be higher for interventions that offer exclusive resources such as in-person interventions, group therapy, or supervised exercise classes that provide access to equipment, facilities, and expert guidance that individuals may find valuable.

Beliefs, Attitudes, and Habits

Beliefs about body weight can also significantly affect an individual's WTP for weight loss interventions. Individuals with negative beliefs about their weight and body image may be more motivated to invest in weight loss interventions [1,2,10]. They may also be more likely to experience negative consequences related to their weight, such as discrimination, health problems, or low self-esteem, which in turn can motivate them to take action.

Attitudes can significantly determine an individual's willingness to pay for obesity interventions (10). Understanding these attitudes is crucial for designing effective interventions and communication strategies that resonate with the individual, as the perceived importance of obesity may drive the motivation to invest in solutions.

Habits are deeply ingrained behaviors that people engage in regularly and often without conscious thought and can both facilitate and hinder WTP. People with established healthy habits may be more inclined to invest in obesity interventions [10]. Conversely, individuals with solid habits of unhealthy lifestyles may resist investing in interventions that require significant dietary changes.

It is important for weight loss interventions to address both the physical and psychological aspects to ensure that individuals can achieve their optimum weight loss goals in a sustainable and healthy manner.

## Conclusions

WTP for weight loss interventions is directly associated with being female, increasing BMI, age, perceived health, income, co-morbidities, education levels, percentage of weight loss, long-term health risk reduction, time to noticeable weight loss, delivery mode of interventions, side effects, lifestyle modification, beliefs, attitudes, habits, and cost. It is important to consider these factors when developing weight loss interventions and pricing strategies, especially for groups with lower WTP. By understanding the varying WTP across different sociodemographic groups, unique initiatives can be tailored to meet the needs of specific populations, particularly the sub-groups with low WTP. The interventions will then be priced accordingly to ensure accessibility for all, which is a priceless strategy for reducing the incidence of obesity at the local and global levels.

The identification of common attributes of the WTP for overweight and obesity intervention can assist in the formulation and implementation of effective evidence-based policies. Specific sub-groups with low WTP could be targeted via unique initiatives to improve their participation in weight-loss interventions. This will ensure that no groups are marginalized in the fight against the global obesity epidemic. This review provides valuable insights into various aspects of associated attributes of WTP to benefit financial decision-making to prioritize public health interventions. There variations in the study designs, methods, target populations, study duration, and perspectives across the studies in this systematic review provide alternatives in various interventions that can be tailored to the associate attributes for the local populations to be accepted and applied.
